# Health-Related Social Needs during the COVID-19 Pandemic: The Chronic Renal Insufficiency Cohort (CRIC) Study

**DOI:** 10.34067/KID.0000000000000439

**Published:** 2024-04-15

**Authors:** Tessa K. Novick, Michelle Osuna-Diaz, Lawrence J. Appel, Jeanne B. Charleston, James P. Lash, Natalie Meza, Debbie L. Cohen, Angela Allen, Deidra C. Crews, the CRIC Study Investigators

**Affiliations:** 1 Dell Medical School, University of Texas at Austin, Austin, Texas; 2 Welch Center for Prevention, Epidemiology, and Clinical Research, Johns Hopkins Bloomberg School of Public Health, Baltimore, Maryland; 3 Department of Medicine, University of Illinois, Chicago, Illinois; 4 Center for Clinical Epidemiology and Biostatistics, Perelman School of Medicine at the University of Pennsylvania, Philadelphia, Pennsylvania; 5 Johns Hopkins Center for Health Equity, Johns Hopkins Medical Institutions, Baltimore, Maryland; 6 Division of Nephrology, Department of Medicine, Johns Hopkins University School of Medicine, Baltimore, Maryland

**Keywords:** CKD, health equity, diversity, and inclusion

Health-related social needs among people with CKD are associated with risk factor control and delays in seeking medical care.^1–3^ Social needs were exacerbated for many during the coronavirus disease 2019 (COVID-19) pandemic. Our objective was to assess the prevalence of needs and identify correlates among a cohort of people with CKD during the COVID-19 pandemic.

The Chronic Renal Insufficiency Cohort (CRIC) study is a multicenter cohort of nearly 5500 adults with CKD.^4,5^ CRIC participants undergo annual in-person and telephone visits every 6 months. Questionnaires were administered to participants from three sites from 2020 to 2023, chosen to optimize sample size and diversity, and included questions about housing, food, transportation and utility needs. To be consistent with national screening efforts, the questions were taken from the Centers for Medicare & Medicaid Services Accountable Health Communities Health-related Social Needs Screening Tool.^6^ We performed a cross-sectional analysis of 632 adults with non–dialysis-dependent CKD. Participants completed the questionnaire by phone, in English or Spanish. The institutional review board at each study site approved the study.

We defined housing needs as an individual being worried about losing housing; not having a steady place to live; or problems with pests, mold, lead, heat, ovens, smoke detectors or water. We defined food needs as an individual reporting they sometimes/often worried that food would run out before getting money for more or that their food did not last and money was unavailable to buy more. We defined transportation needs as an individual reporting that lack of reliable transportation kept them from necessary activities. We defined utility needs as an individual reporting their electric, gas, oil, or water company threatened to shut off services in their home.

We examined the prevalence of screening positive for at least one need and of each individual need. We compared characteristics according to whether someone screened positive for at least one need, and we displayed data as mean (SD) for continuous variables and number and percentages for categorical variables. We used logistic regression with all covariates included in each model to identify correlates of needs. We performed a separate logistic regression analysis for each outcome, including screening positive for at least one need and housing, food, transportation, and utility needs.

Among 632 participants, 50% were female, the mean age was 72 years, and 40% identified as Black and 26% identified as Hispanic. There were 192 participants (30%) who screened positive for at least one need, among whom 123 (19%), 70 (11%), 74 (12%), and 26 (4%) screened positive for housing, food, transportation, and utility needs, respectively. Compared with participants without needs, those reporting ≥1 need were younger (mean age 69 versus 72), more likely to be female (60% versus 46%), more likely to be Black (45% versus 37%), more likely to be Hispanic/Latin (33% versus 23%), have higher systolic BP (mean 132.2 versus 128.5 mm Hg), and less likely to report obtaining a college education (27% versus 42%).

Variables independently associated with reporting at least one need included age (odds ratio [OR], 0.81; 95% confidence interval [CI], 0.67 to 0.97), Black race (OR, 1.76; 95% CI, 1.07 to 2.90), and education level (OR, 0.55; 95% CI, 0.31 to 0.98 for a college degree versus less than a high school degree) (Table 1).

**Table 1:** Odds ratios associated with reporting ≥1 health-related social need and each individual need

Variable	OR (95% CI) for ≥1 HRSN	OR (95% CI) for Housing Needs	OR (95% CI) for Food Needs	OR (95% CI) for Transportation Needs	OR (95% CI) for Utility Needs
Age, per 10 yr	0.81 (0.67 to 0.97)	0. 72 (0.59 to 0.89)	0.69 (0.53 to 0.89)	0.80 (0.62 to 1.02)	0.90 (0.61 to 1.34)
**Sex**					
Male	Ref	Ref	Ref	Ref	Ref
Female	1.48 (0.95 to 2.30)	1.02 (0.61 to 1.70)	2.08 (1.08 to 4.01)	2.57 (1.35 to 4.91)	1.41 (0.53 to 3.73)
**Race/ethnicity**					
Non-Hispanic/Latin White	Ref	Ref	Ref	Ref	Ref
Non-Hispanic/Latin Black	1.76 (1.07 to 2.90)	1.78 (1.01 to 3.13)	3.47 (1.32 to 9.13)	1.81 (0.84 to 3.93)	2.16 (0.44 to 10.55)
Hispanic/Latin	1.71 (0.94 to 3.10)	1.08 (0.53 to 2.18)	5.35 (1.87 to 15.29)	1.71 (0.69 to 4.25)	3.15 (0.59 to 16.93)
Other	1.76 (0.43 to 7.23)	1.36 (0.27 to 6.92)	NA	7.15 (1.51 to 33.74)	NA
**Education level**					
Less than high school	Ref	Ref	Ref	Ref	Ref
High school graduate	0.47 (0.26 to 0.86)	0.58 (0.28 to 1.19)	0.52 (0.23 to 1.21)	0.66 (0.29 to 1.51)	0.58 (0.16 to 2.10)
Some college	0.76 (0.45 to 1.29)	1.09 (0.59 to 2.02)	0.57 (0.27 to 1.19)	0.94 (0.46 to 1.93)	1.26 (0.46 to 3.43)
College graduate	0.55 (0.31 to 0.98)	0.90 (0.45 to 1.77)	0.73 (0.32 to 1.66)	0.62 (0.26 to 1.44)	0.12 (0.01 to 1.05)
Current smoker	1.27 (0.61 to 2.67)	1.36 (0.61 to 3.05)	1.82 (0.71 to 4.64)	0.76 (0.25 to 2.33)	0.61 (0.08 to 4.88)
BP ≥130/80 mm Hg	1.20 (0.84 to 1.72)	1.50 (0.99 to 2.27)	1.53 (0.89 to 2.62)	1.56 (0.93 to 2.63)	1.03 (0.45 to 2.33)
Hemoglobin A1C, per 1%	1.00 (0.87 to 1.15)	0.92 (0.78 to 1.09)	0.92 (0.75 to 1.14)	1.04 (0.86 to 1.26)	1.09 (0.84 to 1.42)
eGFR, per 10 ml/min per 1.73 m^2^	1.02 (0.92 to 1.12)	1.05 (0.94 to 1.17)	0.95 (0.83 to 1.09)	0.96 (0.84 to 1.09)	0.91 (0.73 to 1.14)

Variables were tested in combination with all other variables. There were 632 individuals in analyses for ≥1 health-related social need, housing and transportation needs, and there were 621 individuals in the analyses for food and utility needs. CI, confidence interval; HRSN, health-related social need; NA, not applicable (there were not enough individuals within this category); OR, odds ratio.

Variables independently associated with housing needs included age (OR, 0.72; 95% CI, 0.59 to 0.89) and Black race (OR, 1.78; 95% CI, 1.01 to 3.13). Variables independently associated with food needs included age (OR, 0.69; 95% CI, 0.53 to 0.89), female sex (OR, 2.08; 95% CI, 1.08 to 4.01), Black race (OR, 3.47; 95% CI, 1.32 to 9.13), and Hispanic/Latin ethnicity (OR, 5.35; 95% CI, 1.87 to 15.30).

Variables independently associated with transportation needs included female sex (OR, 2.57; 95% CI, 1.35 to 4.91) and “other” race (OR, 7.14; 95% CI, 1.51 to 33.74). There were no associations between utility needs and other variables.

The findings were similar to nationwide estimates.^7^ Among 1,020,864 Medicare and Medicaid beneficiaries screened for needs between 2018 and 2021, 37% reported at least one need, among whom 68% and 52% experienced food and housing needs, respectively.^7^

Limitations of our study include modest sample size, lack of detailed data on sex, race, and prepandemic needs. Findings may underestimate the prevalence of social needs among people with CKD in the United States and may have been underpowered. Strengths include being among the first to evaluate needs in a national cohort of people with CKD during the COVID-19 pandemic.

Among 632 adults with non–dialysis-dependent CKD in CRIC, we found that 30% experienced at least one need during the COVID-19 pandemic, and people who were younger, female, Black, and/or Hispanic/Latin were at greatest risk. Additional research is needed to understand the scope of needs among people with CKD in comparison with the general population. Screening for needs should be incorporated into routine practice to identify those who are at risk and who might benefit from intervention and linkage with community resources.

## Supplementary Material

## Data Availability

Partial restrictions to the data and/or materials apply. Part of a national cohort (CRIC).
