# Supplementary material for: Health-Related Social Needs during the COVID-19 Pandemic: The Chronic Renal Insufficiency Cohort (CRIC) Study
Source: Kidney360. 2024 Apr 15;5(6):900–2. doi: 10.34067/KID.0000000000000439 (PMC11219102; doi:10.34067/KID.0000000000000439)
Supplement: Supplementary file 1 [file kidney360-5-900-s001.pdf]

ASN American Society of Nephrology | JASN Journal of the American Society of Nephrology | KIDNEY360 Accessing Our World From Every Angle | CJASN Clinical Journal of the American Society of Nephrology

ASN Journal Disclosure Form

As per ASN journal policy, I have disclosed any financial relationship or commitment held by myself and/or my spouse/partner in the past 36 months as included below. I have listed my Current Employer below to indicate there is a relationship requiring disclosure. If no relationship exists, my Current Employer is not listed.

A. Allen reports the following:
Employer: University of Pennsylvania

I understand that the information above will be published within the journal article, if accepted, and that failure to comply and/or to accurately and completely report the potential financial conflicts of interest could lead to the following: 1) Prior to publication, article rejection, or 2) Post-publication, sanctions ranging from, but not limited to, issuing a correction, reporting the inaccurate information to the authors' institution, banning authors from submitting work to ASN journals for varying lengths of time, and/or retraction of the published work.

Name: Angela Allen
Manuscript ID: K360-2023-000878R1
Manuscript Title: Health-Related Social Needs During the COVID-19 Pandemic: the Chronic Renal Insufficiency Cohort (CRIC) Study
Date of Completion: March 5, 2024
Disclosure Updated Date: May 24, 2023

ASN American Society of Nephrology | JASN Journal of the American Society of Nephrology | KIDNEY360 Accessing Our World From Every Angle | CJASN Clinical Journal of the American Society of Nephrology

ASN Journal Disclosure Form

As per ASN journal policy, I have disclosed any financial relationship or commitment held by myself and/or my spouse/partner in the past 36 months as included below. I have listed my Current Employer below to indicate there is a relationship requiring disclosure. If no relationship exists, my Current Employer is not listed.

L. Appel reports the following:
Employer: Johns Hopkins University; Consultancy: Wolters Kluwer for chapters in UpToDate; Honoraria: Wolters Kluwer; and Other Interests or Relationships: Bloomberg Philanthropies.

I understand that the information above will be published within the journal article, if accepted, and that failure to comply and/or to accurately and completely report the potential financial conflicts of interest could lead to the following: 1) Prior to publication, article rejection, or 2) Post-publication, sanctions ranging from, but not limited to, issuing a correction, reporting the inaccurate information to the authors' institution, banning authors from submitting work to ASN journals for varying lengths of time, and/or retraction of the published work.

Name: Lawrence J. Appel
Manuscript ID: K360-2023-000878R1
Manuscript Title: Health-Related Social Needs During the COVID-19 Pandemic: the Chronic Renal Insufficiency Cohort (CRIC) Study
Date of Completion: March 7, 2024
Disclosure Updated Date: May 17, 2023

ASN American Society of Nephrology JASN Journal of the American Society of Nephrology KIDNEY360 Accessing Our World From Every Angle CJASN Clinical Journal of the American Society of Nephrology

ASN Journal Disclosure Form

As per ASN journal policy, I have disclosed any financial relationship or commitment held by myself and/or my spouse/partner in the past 36 months as included below. I have listed my Current Employer below to indicate there is a relationship requiring disclosure. If no relationship exists, my Current Employer is not listed.

J. Charleston reports the following:
Employer: Johns Hopkins University

I understand that the information above will be published within the journal article, if accepted, and that failure to comply and/or to accurately and completely report the potential financial conflicts of interest could lead to the following: 1) Prior to publication, article rejection, or 2) Post-publication, sanctions ranging from, but not limited to, issuing a correction, reporting the inaccurate information to the authors' institution, banning authors from submitting work to ASN journals for varying lengths of time, and/or retraction of the published work.

Name: Jeanne Charleston
Manuscript ID: K3602023000878R1
Manuscript Title: Health-Related Social Needs During the COVID-19 Pandemic: the Chronic Renal Insufficiency Cohort (CRIC) Study," submitted to the Kidney360.
Date of Completion: March 27, 2024
Disclosure Updated Date: March 27, 2024

ASN American Society of Nephrology | JASN Journal of the American Society of Nephrology | KIDNEY360 Accessing Our World From Every Angle | CJASN Clinical Journal of the American Society of Nephrology

ASN Journal Disclosure Form

As per ASN journal policy, I have disclosed any financial relationship or commitment held by myself and/or my spouse/partner in the past 36 months as included below. I have listed my Current Employer below to indicate there is a relationship requiring disclosure. If no relationship exists, my Current Employer is not listed.

D. Cohen reports the following:
Employer: University of Pennsylvania; Consultancy: Medtronic, Recor, novartis; Ownership Interest: incyte - spouse; Research Funding: Medtronic; Recor, NIDDK CRIC study; Honoraria: Medtronic, Recor, novartis; Patents or Royalties: incyte - spouse; Advisory or Leadership Role: spouse - kura oncology; and Speakers Bureau: Medtronic.

I understand that the information above will be published within the journal article, if accepted, and that failure to comply and/or to accurately and completely report the potential financial conflicts of interest could lead to the following: 1) Prior to publication, article rejection, or 2) Post-publication, sanctions ranging from, but not limited to, issuing a correction, reporting the inaccurate information to the authors' institution, banning authors from submitting work to ASN journals for varying lengths of time, and/or retraction of the published work.

Name: Debbie L. Cohen
Manuscript ID: K360-2023-000878R1
Manuscript Title: Health-Related Social Needs During the COVID-19 Pandemic: the Chronic Renal Insufficiency Cohort (CRIC) Study
Date of Completion: February 5, 2024
Disclosure Updated Date: February 5, 2024

ASN American Society of Nephrology | JASN Journal of the American Society of Nephrology | KIDNEY360 Accessing Our World From Every Angle | CJASN Clinical Journal of the American Society of Nephrology

ASN Journal Disclosure Form

As per ASN journal policy, I have disclosed any financial relationship or commitment held by myself and/or my spouse/partner in the past 36 months as included below. I have listed my Current Employer below to indicate there is a relationship requiring disclosure. If no relationship exists, my Current Employer is not listed.

D. Crews reports the following:
Employer: Johns Hopkins University; Consultancy: Yale New Haven Health Services Corporation Center for Outcomes Research and Evaluation (CORE); Research Funding: Somatus, Inc.; Baxter International; Advisory or Leadership Role: Editorial Board--Journal of Renal Nutrition, Clinical Journal of the American Society of Nephrology, Journal of the American Society of Nephrology; Advisory Group, Health Equity Collaborative, Partner Research for Equitable System Transformation after COVID-19 (PRESTAC), Optum Labs; and Other Interests or Relationships: Executive Councilor, American Society of Nephrology.

I understand that the information above will be published within the journal article, if accepted, and that failure to comply and/or to accurately and completely report the potential financial conflicts of interest could lead to the following: 1) Prior to publication, article rejection, or 2) Post-publication, sanctions ranging from, but not limited to, issuing a correction, reporting the inaccurate information to the authors' institution, banning authors from submitting work to ASN journals for varying lengths of time, and/or retraction of the published work.

Name: Deidra C. Crews
Manuscript ID: K360-2023-000878R1
Manuscript Title: Health-Related Social Needs During the COVID-19 Pandemic: the Chronic Renal Insufficiency Cohort (CRIC) Study
Date of Completion: February 6, 2024
Disclosure Updated Date: January 10, 2024

ASN American Society of Nephrology | JASN Journal of the American Society of Nephrology | KIDNEY360 Accessing Our World From Every Angle | CJASN Clinical Journal of the American Society of Nephrology

ASN Journal Disclosure Form

As per ASN journal policy, I have disclosed any financial relationship or commitment held by myself and/or my spouse/partner in the past 36 months as included below. I have listed my Current Employer below to indicate there is a relationship requiring disclosure. If no relationship exists, my Current Employer is not listed.

J. Lash reports the following:
Employer: University of Illinois at Chicago; and Advisory or Leadership Role: Kidney360.

I understand that the information above will be published within the journal article, if accepted, and that failure to comply and/or to accurately and completely report the potential financial conflicts of interest could lead to the following: 1) Prior to publication, article rejection, or 2) Post-publication, sanctions ranging from, but not limited to, issuing a correction, reporting the inaccurate information to the authors' institution, banning authors from submitting work to ASN journals for varying lengths of time, and/or retraction of the published work.

Name: James P. Lash
Manuscript ID: K360-2023-000878R1
Manuscript Title: Health-Related Social Needs During the COVID-19 Pandemic: the Chronic Renal Insufficiency Cohort (CRIC) Study,
Date of Completion: February 6, 2024
Disclosure Updated Date: March 4, 2023

ASN American Society of Nephrology
JASN Journal of the American Society of Nephrology
KIDNEY360 Accessing Our World From Every Angle
CJASN Clinical Journal of the American Society of Nephrology

ASN Journal Disclosure Form

As per ASN journal policy, I have disclosed any financial relationship or commitment held by myself and/or my spouse/partner in the past 36 months as included below. I have listed my Current Employer below to indicate there is a relationship requiring disclosure. If no relationship exists, my Current Employer is not listed.

N. Meza reports the following:
Employer: University of Illinois at Chicago; and Patents or Royalties: UIC Midwest Latino Health Research Training and Policy Center.

I understand that the information above will be published within the journal article, if accepted, and that failure to comply and/or to accurately and completely report the potential financial conflicts of interest could lead to the following: 1) Prior to publication, article rejection, or 2) Post-publication, sanctions ranging from, but not limited to, issuing a correction, reporting the inaccurate information to the authors' institution, banning authors from submitting work to ASN journals for varying lengths of time, and/or retraction of the published work.

Name: Natalie Meza
Manuscript ID: K360-2023-000878R1
Manuscript Title: Health-Related Social Needs During the COVID-19 Pandemic: the Chronic Renal Insufficiency Cohort (CRIC) Study
Date of Completion: February 27, 2024
Disclosure Updated Date: February 27, 2024

ASN American Society of Nephrology JASN Journal of the American Society of Nephrology KIDNEY360 Accessing Our World From Every Angle CJASN Clinical Journal of the American Society of Nephrology

ASN Journal Disclosure Form

As per ASN journal policy, I have disclosed any financial relationship or commitment held by myself and/or my spouse/partner in the past 36 months as included below. I have listed my Current Employer below to indicate there is a relationship requiring disclosure. If no relationship exists, my Current Employer is not listed.

T. Novick reports the following:
Employer: University of Texas at Austin Dell Medical School

I understand that the information above will be published within the journal article, if accepted, and that failure to comply and/or to accurately and completely report the potential financial conflicts of interest could lead to the following: 1) Prior to publication, article rejection, or 2) Post-publication, sanctions ranging from, but not limited to, issuing a correction, reporting the inaccurate information to the authors' institution, banning authors from submitting work to ASN journals for varying lengths of time, and/or retraction of the published work.

Name: Tessa Kimberly Novick
Manuscript ID: K360-2023-000878R1
Manuscript Title: Health-Related Social Needs During the COVID-19 Pandemic: the Chronic Renal Insufficiency Cohort (CRIC) Study")
Date of Completion: February 6, 2024
Disclosure Updated Date: August 21, 2023

ASN
American Society of Nephrology
JASN
Journal of the American Society of Nephrology
KIDNEY360
Accessing Our World From Every Angle
CJASN
Clinical Journal of the American Society of Nephrology

ASN Journal Disclosure Form

As per ASN journal policy, I have disclosed any financial relationship or commitment held by myself and/or my spouse/partner in the past 36 months as included below. I have listed my Current Employer below to indicate there is a relationship requiring disclosure. If no relationship exists, my Current Employer is not listed.

M. Osuna-Diaz reports the following:
Employer: University of Texas at Austin - Dell Medical School

I understand that the information above will be published within the journal article, if accepted, and that failure to comply and/or to accurately and completely report the potential financial conflicts of interest could lead to the following: 1) Prior to publication, article rejection, or 2) Post-publication, sanctions ranging from, but not limited to, issuing a correction, reporting the inaccurate information to the authors' institution, banning authors from submitting work to ASN journals for varying lengths of time, and/or retraction of the published work.

Name: Michelle Marie Osuna-Diaz
Manuscript ID: K360-2023-000878R1
Manuscript Title: Health-Related Social Needs During the COVID-19 Pandemic: the Chronic Renal Insufficiency Cohort (CRIC) Study
Date of Completion: February 5, 2024
Disclosure Updated Date: February 5, 2024
